# Exploring the role of religion in the recovery experiences of suicide attempt survivors in Ghana

**DOI:** 10.1186/s12888-023-04674-3

**Published:** 2023-03-30

**Authors:** Joseph Osafo, Winifred Asare-Doku, Charity S. Akotia

**Affiliations:** 1 Department of Psychology, University of Ghana, Legon, Ghana; 2 National Drug and Alcohol Research Centre, University of New South Wales, Sydney, Australia; 3 Centre for Suicide and Violence Research-CSVR, Ghana, CSVR, Accra, Ghana

**Keywords:** Exploring, Meaning, Suicide, Religion, Ghana

## Abstract

**Background:**

Religion performs a doubled edged role in a suicide crisis. On the one hand, it elicits empathic responses towards suicidal persons. On the other, it condemns and shames them. Although there is evidence that religion promotes better health and general wellbeing, little attention has been given to its role in recovery, especially after a suicide attempt. The current study explored how religion facilitated recovery among suicide attempt survivors.

**Methods:**

Using a semi-structured interview guide, we interviewed suicide attempt survivors who had attended a psychiatric unit. Thematic analysis was used to analyse the data.

**Results:**

Ten suicide attempters were interviewed, six females and four males. Three major themes were identified: Reasons in context, Religion in the recovery process and Renewed commitment to religious rituals/practices.

**Conclusions:**

The role of religion in suicide prevention as a resource, is a complex one. Suicide preventionists need to carefully guide and gauge their prevention efforts in context where religion is rife to provide suicide attempt survivors the most effective religious resource in their recovery trajectory.

## Introduction

Evidence shows that religion and spirituality play a major role in the suicidal path [1, 2]. For instance, a study in Ghana by Akotia, Knizek [3] reported that attempted survivors experienced spiritual struggles prior to or during their hospitalisation and when receiving medical attention. This stemmed from the perception that they have sinned against God by attempting suicide and need to seek forgiveness. Thus, the understanding that existential elements (e.g., religion, meaning, etc.) are present in suicidal behaviours, either as recovery resources or risk factors is an important issue in understanding suicide and its prevention in-context [2].

Most religions are concerned with matters of life and death, therefore may have an impact on suicidal behaviour [4]. This reality has been acknowledged by the WHO [5] report on suicide which indicated that religion and spiritual beliefs may offer some protection against suicide. However various studies have presented a double-edged role of religion in suicidality [2]. For instance, while church attendance may protect against suicidality, interpersonal difficulties with church members and beliefs, doubts and fear of punishment from God have been reported to be associated with poor mental health and eventually suicide [4, 6]. Negative spiritual coping also increases suicide risk [7]. Religious affiliation however has been found to protect against suicide attempts but not against suicidal ideation [8]. These inconsistent findings call for further investigations into the role of religion and spirituality in suicidality. The sections below discuss suicidal behaviour, meaning and recovery and its interaction in the context of suicide attempts. It also discusses the literature on the sociocultural factors in Ghana in the context of suicide attempts.

### Suicidal behaviour, meaning and recovery

Suicidal behaviour refers to a range of behaviours including thinking about suicide, planning for suicide, attempting suicide and suicide itself [5]. A suicide attempt is a major risk factor for suicide, it refers to intentional self-inflicted poisoning, injury or self-harm, which may or may not have a fatal intent [5]. It is therefore a stressful situation, especially when the reason for attempting remains unchanged. Individuals find ways to cope with the stressors of life using various coping methods, including religious coping [9]. People embark on a quest to work out the meaning of what is happening to them through religion. They seek to find meaning when experiencing an existential crisis.

Meaning refers to the perceived nature of the relationship between the individual and his/her world developed within the context of specific events [10]. When suicide or suicide attempt occurs, people long to find meaning in what happened and try to understand events surrounding the act. Meaning, therefore, is that which pertains to one’s identity and how that is affected by the suicide attempt; and meaning also is that which pertains to perceived characteristics of the attempt and to the social circumstances that surround it [10]. According to Ryff [11] the “will to meaning” which is constructing meaning from life’s events–is an essential human characteristic, and forms a critical element of psychological well-being. If this quest is unfulfilled or blocked, it can lead to physical and mental discomfort [12]. Some protective or resilience factors can help prevent suicide and people recovering following an attempt. These factors include having a reason to live, and hope, developing positive coping strategies, providing supportive environments, promoting healthy cultural and religious beliefs [8, 13, 14]. These are factors that induce meaning and purpose-driven living, which may be suicide preventive and facilitate recovery after a suicide attempt.

### Relevant socio-cultural contexts of Ghana

A recent study among university students reported the prevalence rates of suicidal behaviours, including suicidal ideations (15.2%), attempted suicide (6.3%), death wishes (24.3%) and having a suicidal plan (6.8%) [15]. Ghana is highly religious [16] but only a handful of studies have examined the role of religion in suicide [3, 17–19]. Consequently, any opportunity to examine the role of religion (herein, Christianity) in suicide is an important exercise to further our understanding of the relationship between two complex human phenomena: religion and suicide in a proscriptive cultural context such as Ghana [20].

Suicidal behaviour is strongly proscribed in Ghana and attitudes continue to be negative towards the act and the suicidal person [21]. There are three levels at which stigma towards suicide is expressed and permeates the Ghanaian culture. The first level is negative attitude from family/community where the suicidal person is seen as an aggressor and antisocial. Second, religious groups see the suicidal person as a sinner and transgressor; and third, in the eyes of the law, the suicidal person is a criminal [22]. Section 57(2) of the Criminal and Other Offences Act, 1960 (Act 29) thus criminalizes the suicide attempt survivor “*A person who attempts to commit suicide commits a misdemeanour”.*

Mental health is underfunded, understaffed and stigmatized in Ghana [23]. There are only 3 psychiatric hospitals, all located in the southern part of the country. Traditional and faith healing exist alongside orthodox practices [24, 25]. People have long looked to their religious faith for strength and support, particularly in difficult times [26]. There is a preponderance of Pentecostal churches providing divinatory consultation [27] or what Asamoah-Gyadu [28] describe as *religious mediation* for supplicants in search of relief from all forms of existential crisis ranging from illness, financial crisis, unemployment, and marital distress [28]. The triumphal theology of such churches creates confidence in sufferers in perpetual search of their services [28]. In some studies, such religious leaders have reported their palliative role in the treatment and recovery of mentally ill or distressed persons [29]. In fact, in a recent study, religious leaders have clearly reported that they play a key role in suicide prevention in Ghana, including creating healing communities, providing lay counselling, providing referrals to mental health professionals, offering prayer and deliverance, providing social support, and inducing hope in persons in suicidal crisis (Osafo et al., 2021).

Religious coping is categorised into positive and negative: Positive religious coping is when people believe that God is with them through their struggles whereas negative religious coping is when people interpret the struggles as punishment from God whiles experiencing tensions within oneself [30]. We are aware that religion can have some negative impacts on a person’s mental health through self-loathing and self-condemnation, and a threat to harmonious living [4, 31]. However, we are also aware that religion promotes better mental health, physical health, and relationship through routes such as providing meaning, guides for living, attachment to God, love, self-control, forgiveness, etc. [4]. This is a delicate balance from which we approach this present study on religion and spirituality and their relationship with mental health (herein, suicide). While some studies in Ghana do report the positive impact of religion on the mental health of supplicants such as connectedness, hope induction, public education and counselling, perceived prayer and supernatural intervention [19, 29, 32]; others have also reported the negative dimension of religion on the mental health of supplicants such as abuse, religion-induced witchcraft accusations, stigma and condemnation following a moral failure of religious persons [33]. Nevertheless, the fact remains, that the potential palliative role of religion in mental health in Ghana has begun to burgeon as important discourse in scaling up cultural resources in mental health services in Ghana [34, 35].

When people engage in suicide and survive, the experience might trigger various existential crises including reconciling their actions with the requirements of their faith and religious dogmas [36]. At the same time, if they live in a religiously rich environment, they might fall on religion to manage their post-suicidal difficulties. This might be a challenge that impedes their recovery. Some evidence for example, in Ghana shows that attempt survivors in Ghana experience tensions with their families, and this makes their post-suicide recovery difficult [37]. In Ghana, religious meetings and participation in church services virtually become extended dimensions of family bonds [38]. An escape from the home following a suicide attempt in search for solace in a healing community from the church may simply be a fruitless endeavour. Although previous studies have examined some coping dimensions of religion along the suicidal path, close attention has not been paid to examining its influence during recovery.

Religion and marriage are some of the key sociological factors whose impact on suicide have been examined at a more macro level than a micro level. In the sense that, when these factors are examined, researchers have only looked at their associations with decrease or increase in suicide rates [39]. Our point of departure in the present study is to go beyond associations and carefully delineate the mechanisms through which religion helps recovery or otherwise, in the life of suicide attempt survivors in Ghana. In this present study, we closely examined the role of religion in suicide attempt survivors. We were interested in exploring how suicide attempt survivors were coping and recovering, and if religion facilitated this recovery, how did that happen?

## Methods

The study was situated at the Department of Psychiatry of the Korle-Bu Teaching Hospital in Accra, Ghana. This is the largest and most popular referral centre in Ghana. Cases were sampled from 40 suicide attempt survivors receiving psychotherapy from the psychiatry unit since 2015. Ten cases met the criteria to be included in the study. Participants were included if the suicide attempt happened in the past 6 months and beyond (Refer to Asare-Doku, Osafo [40] for a detailed methodology). Participants were called using the phone numbers provided on their records. Interested participants were recruited. Of the participants, one person reported being diagnosed with bipolar diagnosis by a psychiatrist. The other participants had no known diagnosis but psychosocial challenges. A semi-structured interview guide was developed based on the literature and administered. The interview guide focused on the reasons for the attempt, coping mechanisms and recovery after the attempt. Some of the items on the interview guide were “How does your relationship to God influence your life”, “Does your suicide attempt have any influence on your relationship to God”, “How does your religion influence your recovery after your attempt?

Participants were informed about the nature of the study, and participants signed the informed consent statements approved by the Ethics Committee for Humanities at the University of Ghana, (Ref. 040/14–15). Refer to Asare-Doku, Osafo [37] for detailed information on ethics process and recruitment of participants. The interviews were conducted by the second author (WAD) which lasted between 40 min to an hour and occurred at a mutually convenient place or the Psychiatry Unit. In all, six males and four females participated in the study. Participants are identified by gender and age. All ten participants reported being religious and Christians with the majority being charismatics and others being orthodox. The majority reported regular attendance at church and participating in religious rituals such as prayer and Bible reading.

### Analysis

Thematic analysis was used to analyse the data deductively. This is one of the most common forms of analysis in qualitative research, emphasizing and identifying patterns within data. This process was done by familiarizing oneself with the data, generating initial codes, searching for themes, reviewing the themes, and naming the themes [41]. The authors collected and transcribed the data and read the data thoroughly. Later, a list of initial codes was generated. These codes were sorted out into potential themes, which were later refined. Each theme was extensively discussed and accepted before moving on with the rest of the themes. This was to ensure the quality and rigour of analysis [42].

As a practising clinical psychologist, WAD was aware that when interviewing the participants one must try and remain neutral, should not influence participants with personal views and reactions, and must listen from the perspective of a researcher. Although difficult, WAD guarded against providing therapy during the interview and directed participants to seek help for their psychological distress.

## Results

Overall, ten participants participated in the study, six were females and four were males aged between 18 and 34 years. The highest educational level reported was tertiary education, and the lowest was junior high school education. Occupations reported included entrepreneurship, dressmaking, transport worker, and students.

Three major themes were identified: *Reasons in context, Religion in the recovery process and Commitment to religious rituals/practices*.

### Reasons in Context

Participants spoke widely about their reasons for the attempt, which ranged from abuse, experiencing a sense of loss and worthlessness, romantic crisis, general distress, suspected diabolism, and having a bipolar disorder.

*My step mum used to beat me and insult me../so one day I took a disinfectant from another woman’s kitchen and drank it and end it all* (F/18)

*The first thing that came to mind before I drank the disinfectant was that, I mean nothing to these people and they don’t appreciate anything I do for them* (M/21)

*I was having a disagreement with my girlfriend over a trivial issue*…*That was the trigger. Already I have a whole lot of back load things that has happened* (M/24)

*I will call it depression and isolation…I had a series of things bothering me…I was not sleeping for a very long time. It just happened...I wanted to die* (F/24)

*I think it was spiritual or something because I wasn’t having any problem with anyone*…*But what really happened that day before I went to take in the medicine, I can’t tell...When I woke up after I was in the hospita*l...( F/21)

These reasons cited by participants are mostly existential crises that led to the suicide attempt. Existential crisis refers to questions about the meaning and purpose of life, and failing to resolve an existential crisis can have serious consequences. In many cases, suicide is linked to feelings of hopelessness and worthlessness and only in one case that having a mental disorder was reported as a risk factor for suicidality. The core of the reasons stated above indicates that all participants were experiencing some form of emotional pain, which led to ending this pain by taking their own lives. For an extensive discussion of these reasons, see Asare-Doku, Osafo [37].

### Religion in the recovery process

This theme focuses on how religion played a critical role in the recovery of suicidal persons after an attempt. Most of the participants sought solace in religion to cope with the stress accompanied by the aftermath of the attempt. They also resorted to religion in meaning-making. A participant expressed his recovery knowing that he is forgiven by God, although he is aware the suicide attempt act is not pleasing to God;

I don’t know what God thinks about it. I do not think he is pleased with me for doing that but since I believe He forgives; I believe he has forgiven me so am hoping I can get closer to him. I want to get closer to him so nothing can draw me away from him. It’s just the kind of questions I ask myself…what’s all this life about, when I die then what? I don’t think about it too much again though…, The only thing I think is, am hoping am here for a reason, am supposed to do something…I think I will be at peace with myself, at least I hope I came here to do something (M, 24)

The above participant was a medical student and reported attempting suicide because of a crisis following the loss of his romantic relationship. The reason for the attempt could be due to other reasons apart from the loss which culminated in the attempt. From his narrative, as indicated above, his recovery appears to be facilitated by a renewed insight into two things: having a positive view of God as forgiving and having insight into his own purpose or meaning in life. Such thinking could create in him a sense of agency as he believes, peace and optimism will no longer elude him in this life. Meaning-making using religion also helped participants to adjust their worldviews to decrease the distress they experienced.

Another participant narrates;

Yes I felt that I had sinned previously (when I attempted) …but through my relationship with the church leaders and encouragement from them I felt better… I became closer to God as I repented, and I know God has forgiven me (F, 34)

This participant’s suicide was also in response to difficulty in a romantic relationship. The role of religion in his recovery took three (3) routes; connectedness and support from religious authorities, repentance which is a shift in orientation from self-destructive behaviours to survival, and a positive perception of God as forgiving.

For example, in the narrative below, religion fostered a new perspective about God in the recovery process:

*“When I was at the hospital the Doctor made a statement that do I know that God has given me a second chance to live, and I realized that what he said was true. Because I was told that when I was being brought to the hospital, I was taken to two or three clinics before I got to Korle Bu hospital so when the Doctor asked and they told him, he was like and I was able to survive? I realized that God really loved me*, *and it wasn’t something that I really wanted to do” (F, 21)*

From the above narrative, religion played a role in her recovery as she received a new view of herself and God after her attempt. From her narrative, her fortuitous survival was an act of divine intervention. The participant was also reassured that God loved her and this might challenge negative self-referential thoughts.

There were instances where participants indicated a contradictory role of religion in the entire suicidal experience as indicated below;

*“My religion is important to me because it makes me get closer to God more. After my suicide experience I knew punishment is there for me for trying to kill myself… so I felt that I had sinned against God.” (18, F).*

This participant’s view of religion is paradoxical. In one breadth, religion fosters attachment to the divine and fosters separation from this attachment for fear of retribution. The latter could be a recipe for spiritual crisis.

### Renewed commitment to religious rituals/practices

Some of the participants narrate of their intensified commitment to some religious rituals and practices following the suicidal experience. It is like a transformed religious energy and awareness of the divine following the suicidal attempt. This helps them to have a closer relationship with God and offers them the opportunity to be of service to their local church:

*“My religion influences my life and makes me feel I’m someone. If you are in Christ, you have everything in this life. Am not sure you will suffer because we are in Christ. Right now it (my religion) pushes me closer to God. So now if I don’t go to church, I feel very bad… I have to pay my tithe, and this Sunday I didn’t go to church so I have it in an envelope, I will pay when I go next week” (M, 21)*

According to the narrative above, the participant finds religion giving him a new energy and orientation which is expressed in the acquisition of a new insight, attachment to the divine and commitment to religious rituals/practices. It is interesting to note that the participant believes she will not suffer because she is *in Christ*^1^ and believes she has everything she needs in life. It begs the question of whether the participant did not have this realisation before the attempt. Regardless, religion was important coping resource.

It is evident in the narrative below that the participant had a relationship with God before the attempt and regardless of the context for the suicide attempt, she did not blame God for the attempt. She explained having a renewed sense of spirituality which may be due to the close encounter with death and surviving.

*“I love God, I believe in Him. I still love Him and fear Him. These days I have been praying very much and things are happening…Its good for me. Each time I go to church I come back really, really motivated…otherwise I would have done things I won’t be proud of. I still have morals and self-control…God influences my life through my faith in him. The positivity, the faith keeps me” ( F, 24).*

It appears from the narrative that the role of religion in her life after her attempt seemed to have heightened. There was an increase in religious rituals (religious commitment), morality, self-regulation through religious ideals and optimism which foster thriving. Religion may prevent suicide through social support provided by one’s religious community.

## Discussion

This study describes the role of religion in recovery and meaning making after a suicide attempt, in the context of various psychosocial stressors. Meaning provides some form of context that is essential to understand and successfully cope with life’s difficulties [10, 18, 43]. In Ghana, religiosity and spiritual coping is utilized as relief from psychosocial stressors [44–46]. Hence it was not surprising that there was a renewed commitment towards religious practices and to God.

The reasons cited in this study are all predominantly related to adversities in living, further confirming that suicidality in Ghana appears to be psychosocially based [3, 36, 47]. In coping with these psychosocial stressors, all participants admitted to turning to religion/spirituality and religious practices after the suicide attempt in their recovery, as seen in the narratives. The religious practices such as increased attendance to church and involvement in church activities provide a support group ready to assist and offer consolation when in distress [48]. It is important to note that actively engaging in religious activities/rituals helped in recovery rather than just having a religious affiliation. Although studies have reported that religiously affiliated people are less likely to have suicidal ideations and attempt [8, 49], active involvement in religious rituals and activities is the strongest evidence [50]. Involvement in religious activities provides an opportunity to develop an extended support network with other members and the clergy, which is also protective against suicide [51].

In all six cases, the attempt survivors have a reconstituted view of God and themselves. They agree that although God has forgiven them, God can also punish them for their sins. In looking inward at themselves, all the participants modified their behaviour in terms of repenting from the act and through that finding meaning and insight into their life’s purpose. Others also sought to repair their relationship with God whiles renewing their behaviour. Making these changes helps them to thrive and have a sense of efficacy in coping with their current circumstance. Consistent with other studies, in crisis situations, spiritual and religious beliefs played a significant role for the survivors in reconstructing themselves, particularly in processes involving identity-negotiating and meaning-making [52]. Thriving by these reconstitutions (God and Self) are helpful in their recovery journey, as survivors may try to reappraise their situation as an opportunity for spiritual growth or rediscover God’s purpose in the entire experience. In addition to religion’s role in recovery, having a strong support system from their core family and friends, medical and mental health professionals, and religious groups cannot be undermined [53].

Furthermore, there appeared to be a renewed sense of living and purpose after the suicide attempt, with the majority attributing it to divine intervention and second chance at living. The suicidal experience seemed to have provided them cognitively, a fresh start mindset to look at the situation as an opportunity to grow and create a better experience in living. Persons with strong religious faith have higher levels of life satisfaction, greater happiness, and fewer negative psychosocial consequences of traumatic life events [4]. Survivors in this study found happiness and satisfaction in leaning towards religion on the road to recovery and engaged in activities like praying and church attendance to develop closeness to God. Relying on spiritual beliefs and engaging in spiritual activities can give hope, strength, and provide meaning during difficult periods. Spirituality, religiousness, and meaning enhance coping, confer hope for the future, provide a heightened sense of control and strength to resist the opportunity to re-attempt, which is needed to initiate and maintain recovery. This appears to be consistent with what the religious leaders in a current study reported, that religious services are critical in suicide prevention programmes in Ghana (Osafo et al., 2021). They accordingly see themselves as frontline workers in Ghana’s quest for improving mental health services and preventing suicide (Osafo et al., 2021).

### Study limitation

The study focused on the role of religion in recovery hence did not explore in-depth other interventions or aspects of recovery which might be beneficial to participants. Regardless of this, evidence shows that the positive aspects of religion facilitate recovery after a suicide attempt, especially for people who are religious. Some participants could not be contacted via the phone numbers provided hence were not recruited. Others lived in another region and hence could not participate in the study due to a lack of funding for travel.

### Implications for clinical practice

This study has many implications for practice in Ghana. The religious context for suicide and recovery is key in Ghana. In handling clinical patients or service users during suicidal crisis, their religious faith could be explored to understand how it complicates or supports the recovery process. Opportunities should be provided for service users broadly to discuss their spirituality or religion in therapy and provide access to religious and spiritual resources. Their religious needs should be explored and assessed throughout the therapy sessions. If this is left unaddressed, it could foster spiritual crisis and suicide risk. Interprofessional collaboration has been found to be helpful in the management of suicide in Ghana (Osafo & Andoh-Arthur, 2021). Consequently, as part of therapy, when assessing suicidal risk, clinicians should take religion into account; they could conduct a spiritual assessment or if incompetent, work together with patients’ religious leaders [20]. It is not uncommon to find religious leaders or chaplains at many health facilities in Ghana, clinicians should thus ensure that all clinical patients are offered the opportunity to speak with a chaplain or religious leader if desired [54]. Religious leaders may help people find meaning in their lives and foster a sense of connectedness among individuals and the community [55]. Osafo [20] advocates building a strong collaboration that will strengthen both the orthodox and traditional/faith healing approaches to health care by harnessing the strengths in each approach. By extension, health facilities could build effective links with religious groups in the local community.

This is an area suicide researchers could study especially in religious/traditional moral cultures to understand cultural resources available to support the recovery of suicide attempters. Evidence shows that if religiosity is experienced as a source of hope and confidence, it reduces the risk of depression in times of mounting stress, facilitates recovery and diminishes suicide risk [48]. The role of religion in recovery during suicidal crisis is viable but requires proper engagement [20, 32]. Further studies are needed to improve our understanding of how religious mechanisms work in the recovery of suicide attempters in Ghana.

In conclusion, religion is central to meaning making when faced with life events. It is also an important resource and coping mechanism which helps people grapple with answers to existential crises to understand the purpose and meaning of what has happened. In this regard, the role of religion in the aftermath of a suicide attempt cannot be underestimated.

## Data Availability

Access to the data can be made available from the corresponding author on reasonable request.

## Footnotes

1 This a popular Christian theological terminology or jargon used to refer to the entire experience of believing in Christ and living the Christian experience.
